# Structure Determination from Single-Molecule X‑ray Scattering Images Using Stochastic Gradient Ascent

**DOI:** 10.1021/acs.jctc.5c00748

**Published:** 2025-08-14

**Authors:** Steffen Schultze, D. Russell Luke, Helmut Grubmüller

**Affiliations:** † 28282 Max Planck Institute for Multidisciplinary Sciences, Am Fassberg 11, 37077 Göttingen, Germany; ‡ Institute for Numerical and Applied Mathematics, 9375 University of Göttingen, Lotzestraße 16-18, 37073 Göttingen, Germany

## Abstract

Scattering experiments using ultrashort X-ray free electron laser pulses have opened a new path for structure determination of a wide variety of specimens, including nanocrystals and entire viruses, approaching atomistic spatial and femtoseconds time resolution. However, random and unknown sample orientations as well as low signal-to-noise ratios have so far prevented a successful application to smaller specimens like single biomolecules. We here present resolution-annealed stochastic gradient ascent (RASTA), a new approach for direct atomistic electron density determination, which utilizes our recently developed rigorous Bayesian treatment of single-particle X-ray scattering. We demonstrate electron density determination at 2 Å resolution of various small proteins from synthetic scattering images with as low as 15 photons per image.

## Graphical abstract

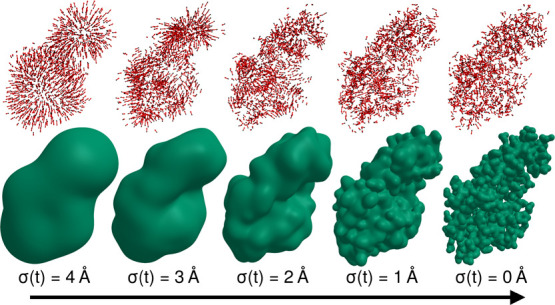

## Introduction

Single-particle X-ray scattering experiments using ultrashort X-ray free electron laser pulses (XFELs) allow for electron density determination approaching atomistic spatial and femtoseconds time resolution.^1–4^ In these ‘diffraction before destruction’^5^ experiments (Figure 1), each sample particle is exposed to a high-intensity X-ray pulse, and the resulting scattering image is recorded on a detector. The femtosecond pulse duration ensures that the scattering outruns the destruction of the sample, which therefore is observed essentially without radiation damage.^6^

**Figure 1:**
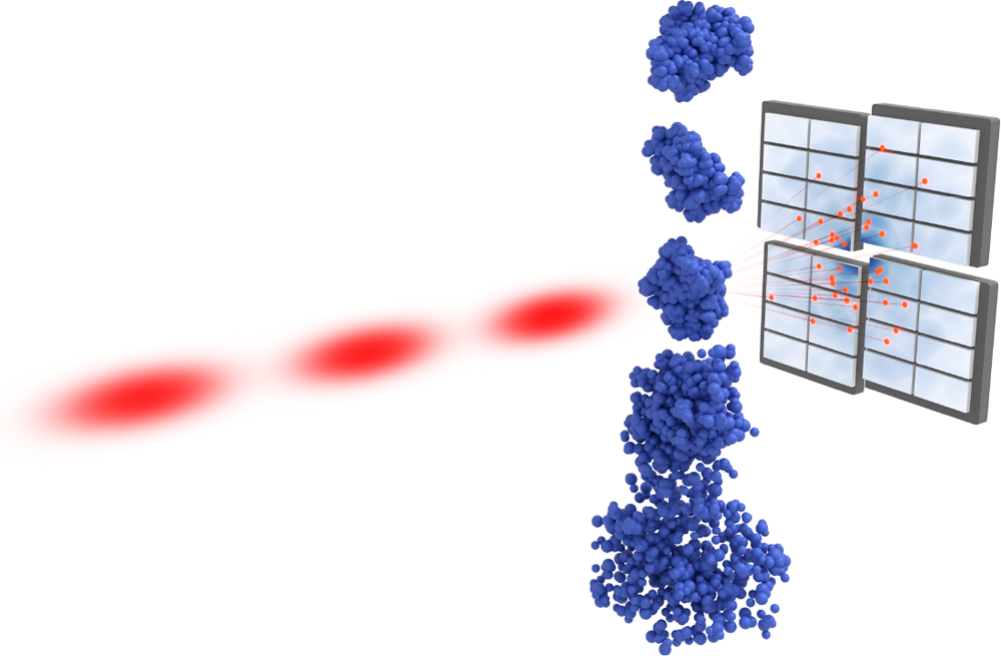
Single molecule scattering experiment.

Many specialized approaches for structure determination from these experiments have been developed, including approaches for orientation determination,^7–14^ manifold embedding,^15–18^ for phase retrieval,^19,20^ or using photon correlations,^21–29^ and have been successfully applied to a broad variety of specimens, including nanocrystals^30–33^ and entire viruses.^34–36^ However, while the idea of performing these experiments also on single macromolecules was proposed over two decades ago, their relatively small size presents formidable challenges, due to the much smaller expected number of typically 10–100 scattered photons per image.^29^ As a consequence of these low photon counts, and in contrast to larger samples, for small proteins it is fundamentally impossible to determine their orientation, which is random and unknown for each image. Most approaches however rely on this orientation information, or, in the case of photon correlations,^21,29^ discard too much information to be applicable at realistic numbers of images.

To circumvent this problem, we have recently developed a rigorous Bayesian treatment^37^ of single-particle X-ray scattering. Rather than attempting to determine the sample orientation for each scattering image, this approach rests on a likelihood function for the full set of scattering images that includes a probability weighted average over all possible molecular orientations. In addition, it allows for systematic inclusion of experimental noise and uncertainties such as background noise, polarization, and incomplete detector coverage. As a further benefit, the electron density is directly determined in real space, thus also circumventing the phase problem. In contrast to orientation determination methods, however, our Bayesian approach does not directly construct an electron density map. Instead, it requires optimizing or sampling from the Bayesian posterior using techniques like Markov chain Monte Carlo (MCMC). As is typical for MCMC, the computational cost increases rapidly for more complex structures, which has so far limited the application of this approach to rather small structures with a few hundred degrees of freedom.

We here present a new technique for efficient calculation of the gradient of the logarithmic posterior, and a new optimization method we call resolution-annealed stochastic gradient ascent (RASTA), which, as we will show, reduces the computational cost of the optimization by a factor of up to 1000. We assess accuracy and efficiency of this approach on a number of synthetic test cases, including the direct determination of all 1300 atomic positions of the protein Lysozyme^38^ from only 10^6^ simulated scattering images.

## Theory and Methods

### Bayesian Formalism

We seek to determine the electron density ρ that maximizes the Bayesian posterior probability P(ρ|I) of the molecular electron density ρ given the *full* set of observed scattering images I . By Bayes’ theorem this posterior is given by the likelihood P(I|ρ) of the images given ρ and the prior *P*(ρ),

P(ρ|I)∝P(I|ρ)P(ρ) 1

The likelihood, in turn, decomposes into a product over all *N* single images,

$$P(I\;|\;\rho )=\prod_{j=1}^{N}P(k_{1}^{\left(j\right)},...,k_{n_{j}}^{\left(j\right)}\;|\;\rho )$$ 2

Here, for each scattering event *j* = 1...*N*, the resulting scattering image is described by the positions of the *n*_*j*_ scattered photons on the detector, given by their scattering vectors **k**_1_^(*j*)^,..., **k**_*n*_*j*__^(*j*)^. The joint distribution of these scattering vectors is described by the single-image likelihood^37^

P(k1,...,kn|ρ)=∫SO(3)exp(−I0∫E|ρ^(Rk)|2dk)∏i=1n|ρ^(Rki)|2dR 3

where *I*_0_ is the incoming beam intensity and *E* denotes the Ewald sphere, which implements the forward model specified by the physics of the scattering event. We here focus on a background-free forward model, including only photons from coherent scattering described by the Fourier transform ρ̂ of the electron density. The unknown molecular orientation, described by a rotation matrix **R**, is assumed to be uniformly distributed and marginalized out by integrating over the rotation group SO(3).

Whereas the Bayesian formalism as presented so far is independent of a specific representation of the electron density ρ, the prior *P*(ρ) is inherently dependent on the atomic positions. Therefore, we use a representation of ρ by a linear combination of Gaussian beads,

$$\rho (r)=\sum_{i=1}^{m}\frac{h_{i}}{(w_{i}\sqrt{2\pi }{)}^{3}}\exp\left(-\frac{1}{2w_{i}^{2}}∥r-y_{i}{∥}^{2}\right)$$ 4

with bead positions **y**_*i*_, heights *h*_*i*_ and widths *w*_*i*_. These beads may both represent single atoms or describe larger structural entities. At the targeted atomistic resolution level one common height *h* = *h*_*i*_ and one common width *w* = *w*_*i*_ is sufficient to represent structures by specifying the positions of the heavy atoms and neglecting the hydrogen atoms. For ease of notation we will omit these parameters in the following, and write ρ_**y**_*i*__(**r**) for the corresponding electron density.

We include two kinds of prior information on the bead positions **y**_*i*_. First, they should not occupy the same position, and second, they should form one connected molecule. Including both of these restrictions, the prior reads

$$-\log\;P(\rho_{y_{i}})=\sum_{i=1}^{m}\sum_{j=1}^{m}f_{1}(∥y_{i}-y_{j}∥)+\sum_{i=1}^{m}f_{3}\left(\sum_{j=1}^{m}f_{2}(∥y_{i}-y_{j}∥)\right)$$ 5

where *f*_1_(∥**y**_*i*_ – **y**_*j*_∥) is a short-range repulsive pair potential, the term ∑_*j*=1_^*m*^ *f*_2_(∥**y**_*i*_ – **y**_*j*_∥) smoothly counts the number of neighbors of each bead within a certain radius, and *f*_3_ is a potential ensuring that this number of neighbors remains above a certain threshold. We refer to the supplement for the exact functional forms of *f*_1_, *f*_2_, and *f*_3_.

### Computational Aspects

Our optimization approach relies on efficient computations of the gradient of the log-likelihood logP(I|ρ) . To this end, first consider the computation of the likelihood.^37^ The integral over **R** in the single-image likelihood from eq 3 is approximated in two steps, as illustrated in Figure 2. First, the degrees of freedom around the *x* and *y* axes are taken into account via a weighted average over orientations **R**_*l*_ of the Ewald sphere with weights *w*_*l*_ for 1 ≤ *l* ≤ *n*_**R**_ (Figure 2a). These orientations were constructed from Lebedev grids as described in the supplement. Second, for the *z*-axis degree of freedom (Figure 2b), corresponding to rotations around the beam axis, the intensity function is evaluated on a polar coordinate grid (blue densities in Figure 2b) for each *l*, setting *I*_*l*,*r*,*s*_ = |ρ̂(**R**_*l*_ **q**_*r*,*s*_)|^2^ where **q**_*r*,*s*_ are the center positions of the grid cells, indexed by 1 ≤ *r* ≤ *n*_*r*_ and 1 ≤ *s* ≤ *n*_*s*_ in the radial and angular direction, respectively. For each photon position **k**_*i*_ (red dots in Figure 2b), let *r*(**k**_*i*_) and *s*(**k**_*i*_) denote the radial and angular index of the corresponding grid cell, respectively. The rotations around the beam axis are then equivalently described as offsets *s′* of the angular grid indices *s*(**k**_*i*_), and are therefore included by summing over all these offsets. Including both of these steps, the approximate single-image likelihood now reads

P(k1,...⁢,kn|ρyi)≈∑l=1nRwlexp(−I0λl)1ns∑s′=1ns∏i=1nIl,r(ki),s(ki)+s′modns 6

Here, the integral over the Ewald sphere *E* in eq 3, which only depends on **R**_*l*_ , is estimated from the same intensity grids as

$$\lambda_{l}=\sum_{r=1}^{n_{r}}\sum_{s=1}^{n_{s}}a_{r,s}I_{l,\;r,s}$$ 7

where *a*_*r*,*s*_ is the area of each grid cells. As a further benefit of the polar intensity maps, the density of grid cells is naturally higher near the origin where the intensity is highest, improving the accuracy of this estimate.

**Figure 2:**
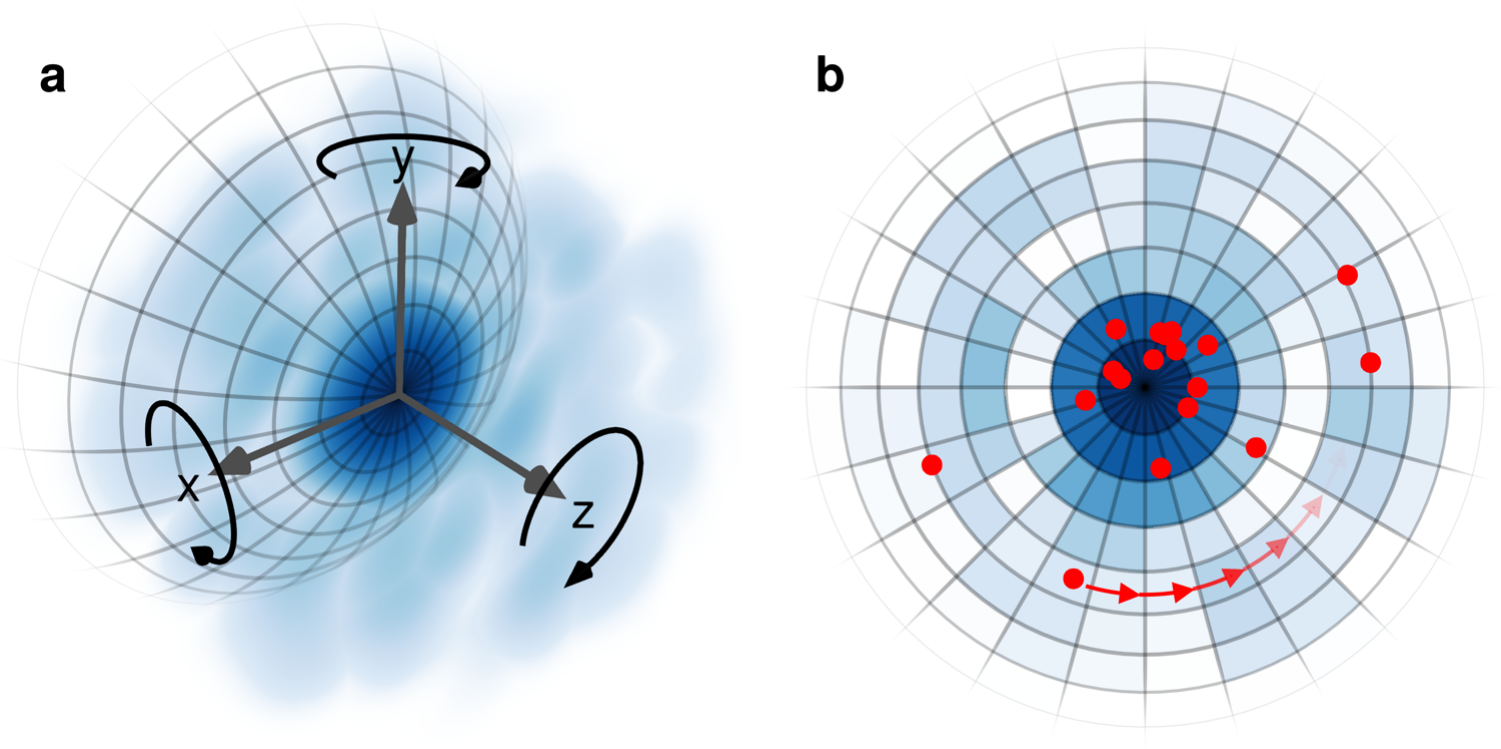
Computation of likelihood. (a) Exemplary orientation of the Ewald sphere (mesh) within the Fourier intensity function *I*(**k**) = |ρ̂(**k**)|^2^ (blue density). (b) Rotations around the beam axis correspond to rotations of the photon positions (red dots) within the intensity maps in polar coordinates (blue density). Shown grid dimensions are reduced for visual clarity.

From the above approximation of the likelihood, the gradient is calculated by backpropagation, that is, by first computing the derivatives with respect to each *I*_*l*,*r*,*s*_ and then applying the chain rule to obtain the gradients with respect to **y**_*i*_, as detailed in the supplement. Both the computation of the likelihood in eq 6 as well as of the gradient were implemented as CUDA-kernels in the Julia programming language,^39^ which we provide as part of our open source software at https://gitlab.gwdg.de/sschult/xfel. Utilizing the special treatment of the beam axis rotations as explained above is essential for performance, as it allows for a much better utilization of GPU-hardware. For instance, we observed a reduction in compute time by about 2 orders of magnitude for the Lysozyme test case below.

### Resolution-Annealed Stochastic Gradient Ascent

A naive application of gradient ascent on the log-posterior $-\log\;P(\rho_{y_{i}}\;|\;I)$ would fail, as it is highly nonconvex and has exceedingly many local minima. Applying Markov-chain Monte Carlo or tempered sampling approaches is similarly challenging due to the exceedingly high number of degrees of freedom. We therefore developed a ‘resolution-annealed stochastic gradient ascent’ (RASTA), which circumvents these minima by removing the high-*k* photons in the beginning of the optimization, and slowly adding them back in over the optimization steps *t*. To this end, at each optimization step *t* we consider a smoothed version $S_{\sigma (t)}(\rho_{y_{i}})=\rho_{y_{i}}*N(0,\sigma (t))$ of the electron density obtained by convolution with a Gaussian kernel N(0,σ(t)) , and exploit the Fourier convolution theorem to obtain a set $S_{\sigma (t)}(I)$ of scattering images corresponding to this smoothed density by rejection sampling. Given an image **k**_1_,..., **k**_*n*_ corresponding to ρ, each photon at position **k**_*i*_ is included in the image corresponding to *S*_σ(*t*)_(ρ) with probability given by exp­(−σ­(*t*)^2^ **k**_*i*_^2^).

Each update is then performed according to the gradient of the log-posterior of *S*_σ(*t*)_(ρ_**y**_*i*__) given $S_{\sigma (t)}(I_{t})$ ,

$$v_{i}(t+1)=\beta (t)v_{i}(t)+\frac{\partial }{\partial y_{i}}\log\;P(S_{\sigma (t)}(I_{t})\;|\;S_{\sigma (t)}(\rho_{y_{i}(t)}))$$ 8

$$y_{i}(t+1)=y_{i}(t)+\eta (t)v_{i}(t+1)$$ 9

where $I_{t}$ denotes a random batch of images used for step *t*. The effect of this smoothing on the gradient can be seen in Figure 3, which shows an exemplary optimization run on the Lysozyme protein (as explained in the results section). As can be seen, the smoothed gradient (Figure 3a) corresponds to large-scale of the smoothed electron density (Figure 3b) updates during the early phase of the optimization and subsequently smaller scale updates as the optimization proceeds. The smoothing scale σ­(*t*) is reduced to zero with increasing *t*, at which point all photons are included. Also the step size η­(*t*) and the momentum parameter β­(*t*) are properly adapted during the optimization, as described in the supplement.

**Figure 3:**
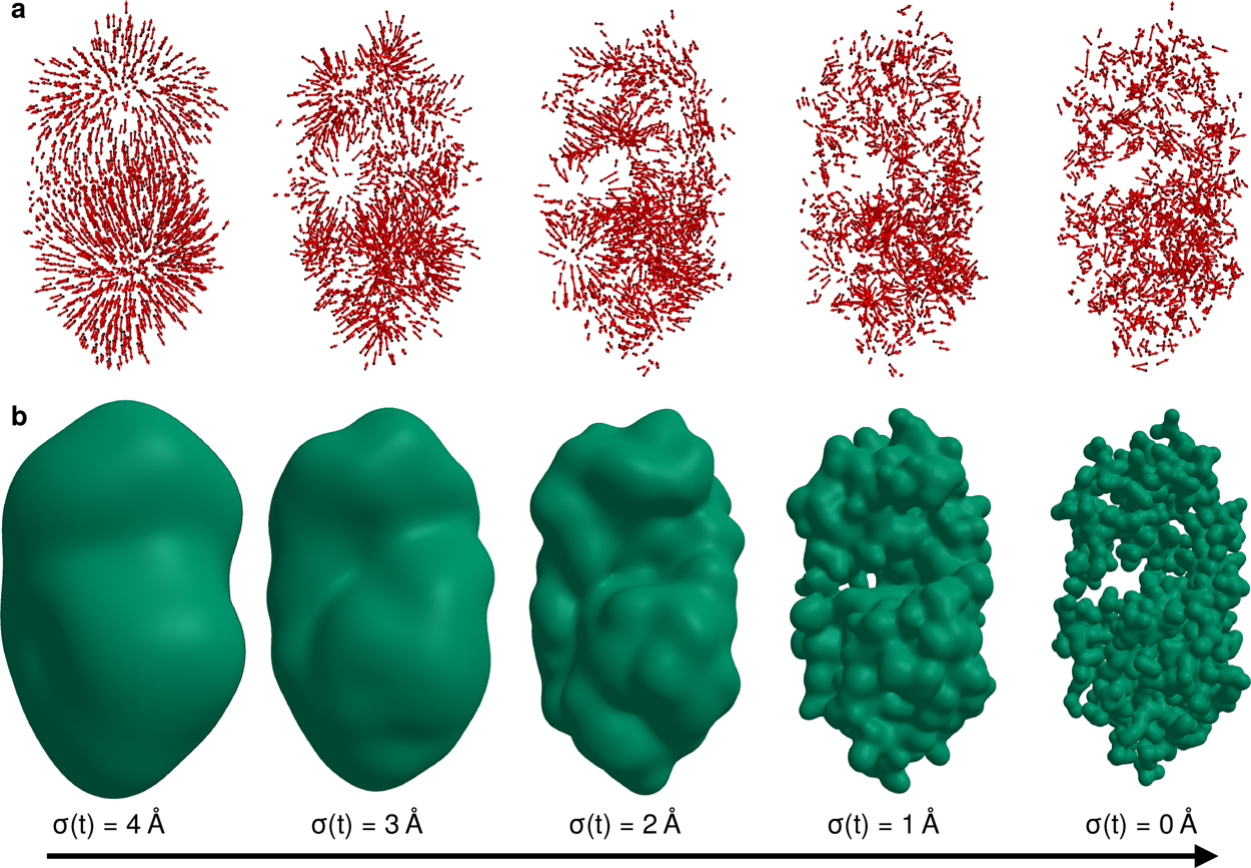
Snapshots from an exemplary optimization run (on the Lysozyme, PDB 148L), at optimization times *t* where σ­(*t*) reaches selected thresholds. (a) Gaussian bead positions (black dots) and current stochastic gradient (red arrows). (b) Electron density snapshots.

This approach is stochastic not only due to the random batches $I_{t}$ of images used for each step,^40^ but also because the rejection sampling to obtain $S_{\sigma (t)}(I)$ has a different random outcome for each step. As a result, although the rejection sampling discards many photons for each single step, with increasing iterations each photon will be included almost surely, such that no information is lost even in the early stages of optimization.

## Results and Discussion

To assess the accuracy, achievable resolution, and computational efficiency of our approach, we selected three small to medium-sized globular proteins as test cases (Table 1), the 46-residue protein Crambin (PDB 1EJG^41^), the 112-residue neuronal nitric oxide synthase PDZ-domain (PDB 1QAU^42^), and the 167-residue protein Lysozyme (PDB 148L^38^). For each case, between 10^6^ and 10^7^ synthetic scattering images were generated as described in the supplement from the reference structure taken from the protein data bank.^43^ Examples of these scattering images are shown in Figure 4a.

**Table 1:** Test Cases

name	PDB	heavy atoms	expected photons per image	number of images
Crambin	1EJG	327	15	10^7^
PDZ-domain	1QAU	812	42	10^6^
Lysozyme	148L	1300	79	10^6^

**Figure 4:**
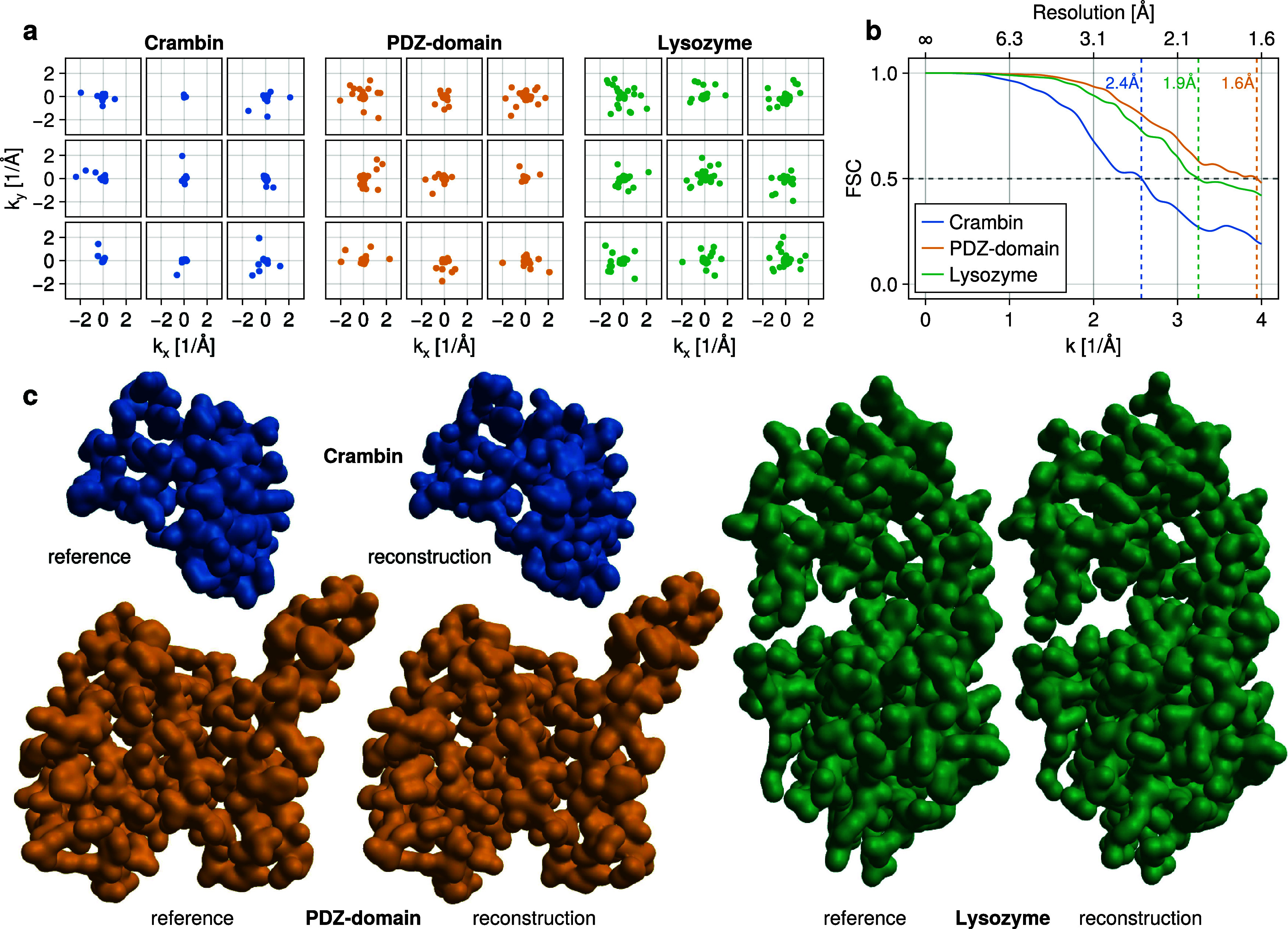
Exemplary application on Crambin, PDZ-domain, and Lysozyme. (a) Exemplary simulated scattering images. Note that the majority photons is scattered to low *k*. (b) Fourier shell correlations show achieved resolutions of around 2 Å. (c) Comparison of reference and reconstructed electron densities.

Note that the images are directly generated as lists of photon positions **k**_1_,..., **k**_*n*_ without any specific detector geometry or pixel size, which should be a good approximation due to the high pixel resolutions of current detectors of up to one Megapixel.^44^ An intensity of 10^12^ photons per pulse was assumed with a beam diameter of 1μ m at a wavelength of λ = 2.5 Å, corresponding, for example, to an estimated average 15 photons per image for Crambin.^29,45^ Photons up to 3 Å^–1^ were included, which, for instance, roughly corresponds to placing a 40 cm × 40 cm detector at about 10 cm distance from the sample.

All reconstructions were performed using one Gaussian bead per heavy atom in the reference structure. Detailed information on optimization parameters, annealing, and step size schedules is provided in the supplement. Convergence rates for stochastic iterations such as these have been determined with respect to the Wasserstein distance in probability measure spaces.^40^ This yields stopping criteria with respect to the Wasserstein distance with error estimates for the distance to the invariant distribution, but monitoring such convergence remains an unresolved challenge. Absent a computationally feasible mathematical stopping criterion, each optimization was run for a predetermined number of steps (50,000 for Crambin and 20,000 for PDZ-domain and Lysozyme), which was chosen large enough that consistent results were obtained. Indeed, as shown below, all runs reached structures close to the corresponding reference structures, indicating sufficient convergence. Also the likelihoods of the reconstructions were consistently at least as good as those of the reference structures.

Figure 4c compares the reference structures with the obtained reconstructions. As can be seen, the atomistic positions are nearly completely recovered, as is corroborated by obtained Fourier-shell-correlation (FSC) resolutions of 2 Å and even higher (Figure 4b). For this resolution estimate, a conservative cutoff of 0.5 was used, using other popular cutoffs the resolutions would be even higher.^46^ As a further measure of quality, the optimal transport plans between the bead positions of reconstructed and reference structures were computed, obtaining earth-mover’s distances of 0.9 Å for Crambin, 0.6 Å for the PDZ-domain, and 0.65 Å for Lysozyme.

Interestingly, despite the 10-times higher number of images, the achieved resolution is lowest for the smallest test protein Crambin. This finding is, at first glance, quite counterintuitive, as one might expect that the reduced number of unknowns would require less data. However, for smaller specimens the images contain fewer photons, and therefore less information. To further investigate this information content, we next asked how the achieved resolution depends on the number of images. To answer this question, we performed a number of independent optimization runs for each test case using varying numbers of images (Figure 5). Figure 5a shows the Fourier-shell-correlation for each run, and Figure 5b the FSC-resolution obtained at the conservative threshold of 0.5.

**Figure 5:**
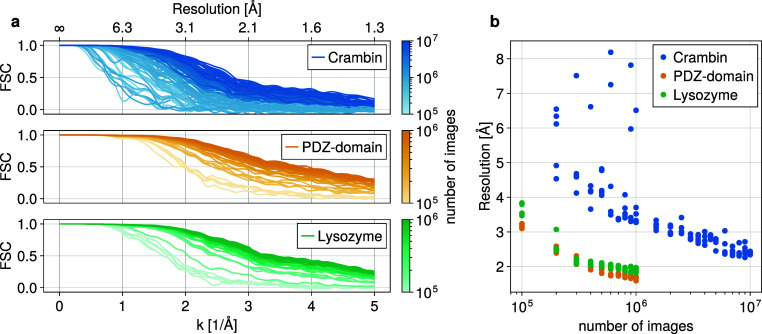
Resolution as a function of the number of images. (a) Fourier shell correlations between reference structure and reconstruction for each test run. (b) Achieved FSC-resolution for each run.

Indeed, for the smallest protein (Crambin) far more images are required to achieve a similar resolution than for the others. For instance, to reliably achieve 2.5 Å resolution, 10^7^ images are required for Crambin, while already 2 · 10^5^ images suffice for the other test proteins. These values differ by a factor of 50, which is much higher than the difference in photon counts per image, showing that the information content per image decreases more than linearly with the number of photons.

It is further notable that the required number of images does not appear to strictly decrease with the number of atoms, either. Indeed, using the same number of images higher resolution is obtained for the PDZ-domain than for the larger Lysozyme. We assume this inversion is due to the less symmetric shape of the PDZ-domain.

## Conclusions

We have developed and assessed a resolution-annealed optimization approach using stochastic gradient descent to determine electron densities at near-atomistic resolution from sparse single-molecule X-ray scattering images.

Compared to previous approaches, the computational effort to achieve high resolution was reduced drastically, making structure determination of small to medium-sized proteins and other biomolecules computationally feasible. Not only is the required compute time highly reduced for any given resolution, but our approach also should also enable one to achieve resolutions that were previously unattainable. For instance, our previous hierarchical MCMC approach^37^ reached computational limits already at 4 Å resolution, requiring more than 1000 GPU-hours in total. In comparison, the implementation of the approach developed here achieves 2 Å requiring only a few GPU-hours, and 4 Å within a few minutes. Further, the computational effort of the approach presented here is independent of the number of images, and only directly depends on the chosen batch size, which will be pivotal for future application to noisy experimental data.

Generalizing this optimization approach to images with background photons in addition to the coherently scattered photons included here, as we have already specified,^37^ is straightforward, although substantially more images will be required. Importantly, our Bayesian approach is also very efficient in terms of the required number of images; in fact, it utilizes the complete available structural information and is therefore in this regard optimal. The comparison to approaches based on photon correlations, which are the only other methods applicable in this extreme Poisson regime, is particularly striking. For instance, whereas the three-photon correlation method^29^ required about 2 · 10^9^ images to achieve a 3.3 Å resolution for Crambin, our tests demonstrated that only 1.5 · 10^6^ suffice to achieve similar resolution. This over thousand-fold improvement will likely translate to more realistic images with background noise.

Here, and in order to explore the maximum achievable resolution under ideal conditions, we focused on a simplified forward model without background noise. Note, however, that our Bayesian framework has already been generalized to take into account experimental effects such as background noise, intensity fluctuations, detector geometry, and polarization.^37^ Applying RASTA to such data will require further testing and tuning of optimization parameters, which is outside of the scope of this paper. In particular, while the Bayesian framework can include detector geometries that exclude low-*k* photons, application of RASTA in that situation will require further testing. Notably, while the direct optimization of electron densities may lead to challenges here, it may also allow easy inclusion of prior information to handle even situations were much of the low-*k* information is missing. For a successful application to experimental scattering data on single molecules it may also be necessary to include further experimental effects, such as low hit rates or preferred orientations. The latter may either be included by weighting the rotational marginalization, or by inferring these weights together with the bead positions.

Although not explored here, we also note that RASTA can serve to obtain uncertainty estimates for the reconstructed electron density by proper sampling of the posterior, for instance in combination with MCMC or Hamiltonian Monte Carlo methods. To what extent this sampling may suffer from the curse of dimensionality, and how it may be overcome, will have to be explored in future work.

From a more general viewpoint, cryogenic electron microscopy (cryo-EM) shares many similarities with single-molecule X-ray scattering, including random and unknown molecule orientations. Although our rejection-sampling based smoothing approach does not have an exact analogue, an similar resolution-annealed stochastic gradient approach should exist, and may be worth exploring in the future.

## Supplementary Material



## Notes

Detailed explanation of gradient computation, definition of prior and info on computational and optimization parameters (PDF)

## References

[1] Hajdu J. (2000). Single-Molecule
X-ray Diffraction. Curr. Opin. Struct. Biol..

[2] Huldt G., Szőke A., Hajdu J. (2003). Diffraction Imaging of Single Particles
and Biomolecules. J. Struct. Biol..

[3] Gaffney K. J., Chapman H. N. (2007). Imaging Atomic Structure
and Dynamics with Ultrafast
X-ray Scattering. Science.

[4] Miao J., Ishikawa T., Robinson I. K., Murnane M. M. (2015). Beyond Crystallography:
Diffractive Imaging Using Coherent x-Ray Light Sources. Science.

[5] Chapman H. N., Caleman C., Timneanu N. (2014). Diffraction before Destruction. Philosophical Transactions of the Royal Society B: Biological
Sciences.

[6] Neutze R., Wouts R., van der
Spoel D., Weckert E., Hajdu J. (2000). Potential
for Biomolecular Imaging with Femtosecond X-ray Pulses. Nature.

[7] Shneerson V. L., Ourmazd A., Saldin D. K. (2008). Crystallography without Crystals.
I. The Common-Line Method for Assembling a Three-Dimensional Diffraction
Volume from Single-Particle Scattering. Acta
Cryst. A.

[8] Loh N.-T. D., Elser V. (2009). Reconstruction
Algorithm for Single-Particle Diffraction
Imaging Experiments. Phys. Rev. E.

[9] Walczak M., Grubmüller H. (2014). Bayesian Orientation
Estimate and Structure Information
from Sparse Single-Molecule x-Ray Diffraction Images. Phys. Rev. E.

[10] Kassemeyer S., Jafarpour A., Lomb L., Steinbrener J., Martin A. V., Schlichting I. (2013). Optimal Mapping of X-Ray Laser Diffraction
Patterns into Three Dimensions Using Routing Algorithms. Phys. Rev. E.

[11] Elser V. (2011). Three-Dimensional
Structure from Intensity Correlations. New J.
Phys..

[12] Tegze M., Bortel G. (2012). Atomic Structure of
a Single Large Biomolecule from
Diffraction Patterns of Random Orientations. J. Struct. Biol..

[13] Flamant J., Le Bihan N., Martin A. V., Manton J. H. (2016). Expansion-Maximization-Compression
Algorithm with Spherical Harmonics for Single Particle Imaging with
x-Ray Lasers. Phys. Rev. E.

[14] Ayyer K., Lan T.-Y., Elser V., Loh N. D. (2016). Dragonfly: An Implementation
of the Expand–Maximize–Compress Algorithm for Single-Particle
Imaging. J. Appl. Crystallogr..

[15] Fung R., Shneerson V., Saldin D. K., Ourmazd A. (2009). Structure from Fleeting
Illumination of Faint Spinning Objects in Flight. Nature Phys..

[16] Schwander P., Giannakis D., Yoon C. H., Ourmazd A. (2012). The Symmetries of Image
Formation by Scattering. II. Applications. Opt.
Express, OE.

[17] Giannakis D., Schwander P., Ourmazd A. (2012). The Symmetries of Image Formation
by Scattering. I. Theoretical Framework. Opt.
Express, OE.

[18] Winter M., Saalmann U., Rost J. M. (2016). Enhancing Scattering Images for Orientation
Recovery with Diffusion Map. Opt. Express, OE.

[19] Elser V., Rankenburg I., Thibault P. (2007). Searching with Iterated Maps. Proc. Natl. Acad. Sci. U. S. A..

[20] Luke D. R. (2005). Relaxed
Averaged Alternating Reflections for Diffraction Imaging. Inverse Problems.

[21] Saldin D. K., Shneerson V. L., Fung R., Ourmazd A. (2009). Structure
of Isolated
Biomolecules Obtained from Ultrashort X-Ray Pulses: Exploiting the
Symmetry of Random Orientations. J. Phys.: Condens.
Matter.

[22] Saldin D. K., Poon H.-C., Schwander P., Uddin M., Schmidt M. (2011). Reconstructing
an Icosahedral Virus from Single-Particle Diffraction Experiments. Opt. Express, OE.

[23] Saldin D. K., Poon H. C., Shneerson V. L., Howells M., Chapman H. N., Kirian R. A., Schmidt K. E., Spence J. C. H. (2010). Beyond Small-Angle
x-Ray Scattering: Exploiting Angular Correlations. Phys. Rev. B.

[24] Saldin D. K., Poon H. C., Bogan M. J., Marchesini S., Shapiro D. A., Kirian R. A., Weierstall U., Spence J. C. H. (2011). New Light on Disordered Ensembles: Ab Initio Structure
Determination of One Particle from Scattering Fluctuations of Many
Copies. Phys. Rev. Lett..

[25] Saldin D. K., Shneerson V. L., Howells M. R., Marchesini S., Chapman H. N., Bogan M., Shapiro D., Kirian R. A., Weierstall U., Schmidt K. E., Spence J. C. H. (2010). Structure of
a Single Particle from Scattering by Many Particles Randomly Oriented
about an Axis: Toward Structure Solution without Crystallization?. New J. Phys..

[26] Starodub D., Aquila A., Bajt S., Barthelmess M., Barty A., Bostedt C., Bozek J. D., Coppola N., Doak R. B., Epp S. W., Erk B., Foucar L., Gumprecht L., Hampton C. Y., Hartmann A., Hartmann R., Holl P., Kassemeyer S., Kimmel N., Laksmono H., Liang M., Loh N. D., Lomb L., Martin A. V., Nass K., Reich C., Rolles D., Rudek B., Rudenko A., Schulz J., Shoeman R. L., Sierra R. G., Soltau H., Steinbrener J., Stellato F., Stern S., Weidenspointner G., Frank M., Ullrich J., Strüder L., Schlichting I., Chapman H. N., Spence J. C. H., Bogan M. J. (2012). Single-Particle
Structure Determination by Correlations of Snapshot X-ray Diffraction
Patterns. Nat. Commun..

[27] Kurta R. P., Donatelli J. J., Yoon C. H., Berntsen P., Bielecki J., Daurer B. J., DeMirci H., Fromme P., Hantke M. F., Maia F. R. N. C., Munke A., Nettelblad C., Pande K., Reddy H. K. N., Sellberg J. A., Sierra R. G., Svenda M., van der Schot G., Vartanyants I. A., Williams G. J., Xavier P. L., Aquila A., Zwart P. H., Mancuso A. P. (2017). Correlations in Scattered X-Ray Laser Pulses Reveal
Nanoscale Structural Features of Viruses. Phys.
Rev. Lett..

[28] Donatelli J. J., Zwart P. H., Sethian J. A. (2015). Iterative Phasing for Fluctuation
X-ray Scattering. Proc. Natl. Acad. Sci. U.
S. A..

[29] von
Ardenne B., Mechelke M., Grubmüller H. (2018). Structure
Determination from Single Molecule X-ray Scattering with Three Photons
per Image. Nat. Commun..

[30] Schlichting I. (2015). Serial Femtosecond
Crystallography: The First Five Years. IUCrJ..

[31] Chapman H. N. (2017). Structure
Determination Using X-Ray Free-Electron Laser Pulses. Methods Mol. Biol..

[32] Oda K., Nomura T., Nakane T., Yamashita K., Inoue K., Ito S., Vierock J., Hirata K., Maturana A. D., Katayama K., Ikuta T., Ishigami I., Izume T., Umeda R., Eguma R., Oishi S., Kasuya G., Kato T., Kusakizako T., Shihoya W., Shimada H., Takatsuji T., Takemoto M., Taniguchi R., Tomita A., Nakamura R., Fukuda M., Miyauchi H., Lee Y., Nango E., Tanaka R., Tanaka T., Sugahara M., Kimura T., Shimamura T., Fujiwara T., Yamanaka Y., Owada S., Joti Y., Tono K., Ishitani R., Hayashi S., Kandori H., Hegemann P., Iwata S., Kubo M., Nishizawa T., Nureki O. (2021). Time-Resolved Serial Femtosecond
Crystallography Reveals Early Structural Changes in Channelrhodopsin. eLife.

[33] Tenboer J., Basu S., Zatsepin N., Pande K., Milathianaki D., Frank M., Hunter M., Boutet S., Williams G. J., Koglin J. E., Oberthuer D., Heymann M., Kupitz C., Conrad C., Coe J., Roy-Chowdhury S., Weierstall U., James D., Wang D., Grant T., Barty A., Yefanov O., Scales J., Gati C., Seuring C., Srajer V., Henning R., Schwander P., Fromme R., Ourmazd A., Moffat K., Van Thor J. J., Spence J. C. H., Fromme P., Chapman H. N., Schmidt M. (2014). Time-Resolved
Serial Crystallography Captures High-Resolution Intermediates of Photoactive
Yellow Protein. Science.

[34] Seibert M. M., Ekeberg T., Maia F. R. N. C., Svenda M., Andreasson J., Jönsson O., Odić D., Iwan B., Rocker A., Westphal D., Hantke M., DePonte D. P., Barty A., Schulz J., Gumprecht L., Coppola N., Aquila A., Liang M., White T. A., Martin A., Caleman C., Stern S., Abergel C., Seltzer V., Claverie J.-M., Bostedt C., Bozek J. D., Boutet S., Miahnahri A. A., Messerschmidt M., Krzywinski J., Williams G., Hodgson K. O., Bogan M. J., Hampton C. Y., Sierra R. G., Starodub D., Andersson I., Bajt S., Barthelmess M., Spence J. C. H., Fromme P., Weierstall U., Kirian R., Hunter M., Doak R. B., Marchesini S., Hau-Riege S. P., Frank M., Shoeman R. L., Lomb L., Epp S. W., Hartmann R., Rolles D., Rudenko A., Schmidt C., Foucar L., Kimmel N., Holl P., Rudek B., Erk B., Hömke A., Reich C., Pietschner D., Weidenspointner G., Strüder L., Hauser G., Gorke H., Ullrich J., Schlichting I., Herrmann S., Schaller G., Schopper F., Soltau H., Kühnel K.-U., Andritschke R., Schröter C.-D., Krasniqi F., Bott M., Schorb S., Rupp D., Adolph M., Gorkhover T., Hirsemann H., Potdevin G., Graafsma H., Nilsson B., Chapman H. N., Hajdu J. (2011). Single Mimivirus Particles Intercepted
and Imaged with an X-ray Laser. Nature.

[35] Ekeberg T., Svenda M., Abergel C., Maia F. R. N. C., Seltzer V., Claverie J.-M., Hantke M., Jönsson O., Nettelblad C., van der Schot G., Liang M., DePonte D. P., Barty A., Seibert M. M., Iwan B., Andersson I., Loh N. D., Martin A. V., Chapman H., Bostedt C., Bozek J. D., Ferguson K. R., Krzywinski J., Epp S. W., Rolles D., Rudenko A., Hartmann R., Kimmel N., Hajdu J. (2015). Three-Dimensional Reconstruction
of the Giant Mimivirus Particle with an X-Ray Free-Electron Laser. Phys. Rev. Lett..

[36] Hosseinizadeh A., Schwander P., Dashti A., Fung R., D’Souza R. M., Ourmazd A. (2014). High-Resolution Structure of Viruses from Random Diffraction
Snapshots. Philosophical Transactions of the
Royal Society B: Biological Sciences.

[37] Schultze S., Grubmüller H. (2024). Bayesian Electron Density Determination from Sparse
and Noisy Single-Molecule X-ray Scattering Images. Science Advances.

[38] Kuroki R., Weaver L. H., Matthews B. W. (1993). A Covalent Enzyme-Substrate Intermediate
with Saccharide Distortion in a Mutant T4 Lysozyme. Science.

[39] Bezanson J., Edelman A., Karpinski S., Shah V. B. (2017). Julia: A Fresh Approach
to Numerical Computing. SIAM Rev..

[40] Luke, D. R. ; Schultze, S. ; Grubmüller, H. Stochastic Algorithms for Large-Scale Composite Optimization: The Case of Single-Shot X-FEL Imaging. 2024, 10.48550/arXiv.2401.13454.

[41] Jelsch C., Teeter M. M., Lamzin V., Pichon-Pesme V., Blessing R. H., Lecomte C. (2000). Accurate Protein Crystallography
at Ultra-High Resolution: Valence Electron Distribution in Crambin. Proc. Natl. Acad. Sci. U.S.A..

[42] Hillier B. J., Christopherson K. S., Prehoda K. E., Bredt D. S., Lim W. A. (1999). Unexpected
Modes of PDZ Domain Scaffolding Revealed by Structure of nNOS-Syntrophin
Complex. Science.

[43] Berman H. M., Westbrook J., Feng Z., Gilliland G., Bhat T. N., Weissig H., Shindyalov I. N., Bourne P. E. (2000). The Protein Data Bank. Nucleic
Acids Res..

[44] Allahgholi A., Becker J., Delfs A., Dinapoli R., Goettlicher P., Greiffenberg D., Henrich B., Hirsemann H., Kuhn M., Klanner R., Klyuev A., Krueger H., Lange S., Laurus T., Marras A., Mezza D., Mozzanica A., Niemann M., Poehlsen J., Schwandt J., Sheviakov I., Shi X., Smoljanin S., Steffen L., Sztuk-Dambietz J., Trunk U., Xia Q., Zeribi M., Zhang J., Zimmer M., Schmitt B., Graafsma H. (2019). The Adaptive Gain Integrating Pixel Detector at the
European XFEL. J. Synchrotron Rad.

[45] Hantke M. F., Ekeberg T., Maia F. R. N. C. (2016). Condor:
A Simulation Tool for Flash
X-ray Imaging. J. Appl. Crystallogr..

[46] van
Heel M., Schatz M. (2005). Fourier Shell Correlation Threshold Criteria. J. Struct. Biol..

