# Supplementary material for: Structure Determination from Single-Molecule X‑ray Scattering Images Using Stochastic Gradient Ascent
Source: J Chem Theory Comput. 2025 Aug 14;21(16):8227–34. doi: 10.1021/acs.jctc.5c00748 (PMC12392449; doi:10.1021/acs.jctc.5c00748)
Supplement: Supplementary file 1 [file ct5c00748_si_001.pdf]

Supporting information for Structure determination from single-molecule X-ray scattering images using stochastic gradient ascent.

Steffen Schultze,[†] D. Russell Luke,[‡] and Helmut Grubmüller[*,†]

†*Max Planck Institute for Multidisciplinary Sciences, Am Fassberg 11, Göttingen, 37077, Germany*

‡*Institute for Numerical and Applied Mathematics, University of Göttingen, Lotzestraße 16-18, Göttingen, 37073, Germany*

E-mail: hgrubmu@mpinat.mpg.de

1 Computation of gradients

The gradient of the log-likelihood was computed by the chain rule, first computing the derivatives with respect to each $I_{l,r,s}$. These derivatives are given by

$$\frac{\partial \log P(\mathcal{I}\,|\,\rho)}{\partial I_{l,r,s}} = \sum_{j=1}^{N} \frac{1}{P(\mathbf{k}_1^{(j)},\ldots,\mathbf{k}_{n_j}^{(j)}\,|\,\rho)} \frac{\partial P(\mathbf{k}_1^{(j)},\ldots,\mathbf{k}_{n_j}^{(j)}\,|\,\rho)}{\partial I_{l,r,s}},$$

and computed by accumulation over the summands in eq. (6) of the main text. To that end, we rewrite eq. (6) as

$$P(\mathbf{k}_1^{(j)},\ldots,\mathbf{k}_{n_j}^{(j)}\,|\,\rho) \approx \sum_{l=1}^{n_{\mathbf{R}}} \sum_{s'=1}^{n_s} p_{ls'j},$$

where

$$p_{l,s',j} = w_l \exp(-I_0\lambda_l)\frac{1}{n_s}\prod_{i=1}^{n_j} I_{l,r(\mathbf{k}_i^{(j)}),s(\mathbf{k}_i^{(j)})+s' \bmod n_s}.$$

Then

$$\frac{\partial P(\mathbf{k}_1^{(j)},\ldots,\mathbf{k}_{n_j}^{(j)}\,|\,\rho)}{\partial I_{l,r,s}} = \sum_{l=1}^{n_{\mathbf{R}}} \sum_{s'=1}^{n_s} \frac{\partial p_{l,s',j}}{\partial I_{l,r,s}},$$

where $\frac{\partial p_{ls'j}}{\partial I_{l,r,s}}$ is obtained using the product rule as

$$\frac{\partial p_{l,s',j}}{\partial I_{l,r,s}} = -a_{r,s} I_0 p_{l,s',j} + \sum_{i=1}^{n} \begin{cases} \frac{p_{l,s',j}}{I_{l,r,s}} & r = r(\mathbf{k}_i^{(j)}) \wedge s = s(\mathbf{k}_i^{(j)}) + s' \bmod n_s \\ 0 & \text{else,} \end{cases}$$

with the first summand taking into account the dependency of λ_l on $I_{l,r,s}$ via eq. (7) of the main text. Finally, the derivatives with respect to the Gaussian bead positions $\mathbf{y}_i$ are obtained by the chain rule as

$$\frac{\partial}{\partial \mathbf{y}_i} \log P(\mathcal{I}\,|\,\rho) = \frac{\partial I_{l,r,s}}{\partial \mathbf{y}_i} \frac{\partial \log P(\mathcal{I}\,|\,\rho)}{\partial I_{l,r,s}}.$$

Taking the Fourier transform of eq. (4), $I_{l,r,s}$ is given by

$$I_{l,r,s} = |\hat{\rho}(\mathbf{R}_l\mathbf{q}_{r,s})|^2 = \left| \sum_{i=1}^{m} h_i \exp\left(-\frac{w_i^2\|\mathbf{q}_{r,s}\|^2}{2}\right) \exp\left(\mathrm{i}(\mathbf{R}_l\mathbf{q}_{r,s})\cdot\mathbf{y}_i\right) \right|^2$$

where $\mathbf{q}_{r,s}$ are the centers of the polar grid cells as defined in the main text. Note that the $\mathbf{q}_{r,s}$ are represented in Cartesian coordinates and merely arranged on a polar grid. Finally, the gradient of $I_{l,r,s}$ is obtained as,

$$\begin{aligned}\frac{\partial I_{l,r,s}}{\partial \mathbf{y}_i} &= 2\Re\left(\overline{\hat{\rho}(\mathbf{q}_{r,s})}\frac{\partial \hat{\rho}(\mathbf{q}_{r,s})}{\partial \mathbf{y}_i}\right)\\ &= 2h_i \mathbf{R}_l \mathbf{q}_{r,s} \exp\left(-\frac{w_i^2\|\mathbf{q}_{r,s}\|^2}{2}\right)\Re\left(\overline{\mathrm{i}\hat{\rho}(\mathbf{q}_{r,s})}\exp\left(\mathrm{i}(\mathbf{R}_l\mathbf{q}_{r,s})\cdot \mathbf{y}_i\right)\right).\end{aligned}$$

as we have derived in equations (55b), (59) and (60) of Ref. 40 of the main text.

2 Definition of prior distribution

The prior distribution $P(\rho_{\mathbf{y}_i})$ was given by

$$-\log P(\rho_{\mathbf{y}_i}) = \sum_{i=1}^{m}\sum_{j=1}^{m} f_1(\|\mathbf{y}_i - \mathbf{y}_j\|) + \sum_{i=1}^{m} f_3\left(\sum_{j=1}^{m} f_2(\|\mathbf{y}_i - \mathbf{y}_j\|)\right),$$

where f_1, f_2 and f_3 are defined as follows in terms of the smoothstep function

$$f(x, x_0, x_1, y_0, y_1) = y_0 + (y_1 - y_0)\,\tilde{f}\left(\frac{x - x_0}{x_1 - x_0}\right), \quad \tilde{f}(x) = \begin{cases} 0 & x < 0, \\ 3x^2 - 2x^3 & 0 \le x \le 1 \\ 1 & x > 1, \end{cases}$$

which smoothly transitions between y_0 and y_1 on the interval from x_0 to x_1. The short range repulsive pair potential was given in terms of its derivative $f_1'(r) = f(r, 0.8, 0.9, s_1, 0) + s_2 \exp(-r^2/(2d(t)^2))$. The neighbor-counting function was given by $f_2(r) = f(r, 5, 10, 1, 0)$, and, finally f_3 was defined in terms of its derivative $f_3'(r) = f(r, 10, 20, s_3, 0)$. The Gaussian component of $f_1(r)$ was included to ensure a uniform distribution of the Gaussian beads at the beginning of the optimization. The parameter $d(t)$ was decreased to zero with increasing t as shown in Figure S1. For the values s_1, s_2, and s_3 see Table S1.

3 Data generation

Given an electron density ρ and a beam intensity I_0, each image was generated directly as a list of scattering vectors by the following rejection sampling procedure. To generate one synthetic scattering image,

- draw the orientation $\mathbf{R} \sim \mathcal{U}(\mathrm{SO}(3))$ from a uniform distribution on the rotation group,
- draw a Poisson-distributed number of attempts $\tilde{n} \sim \mathrm{Pois}\left(I_0 4\pi(2\pi/\lambda)^2 |\hat{\rho}(\mathbf{0})|^2\right)$, where $4\pi(2\pi/\lambda)^2$ is the volume of the Ewald sphere,
- draw photon positions $\mathbf{k}_1, \ldots, \mathbf{k}_{\tilde{n}}$ uniformly distributed on the Ewald sphere, and accept each with probability $|\hat{\rho}(\mathbf{Rk})|^2 / |\hat{\rho}(\mathbf{0})|^2$.

Note that $|\hat{\rho}(\mathbf{k})|^2$ is always maximal at $\mathbf{k} = \mathbf{0}$, such that this procedure works correctly.

4 Optimisation details

Parameter schedules. The optimization parameter schedules for the step size $\eta(t)$, the momentum parameter $\beta(t)$, the smoothing length scale $\sigma(t)$ as well as the repulsive force length scale $d(t)$ are shown in Figure S1. They were determined by trial and error, independently for each test case. Whereas the tests on PDZ-domain and Lysozyme are quite similar (mostly differing by a scale for η and σ, the smallest test protein (Crambin) required a substantially different parameter schedule than the others, due its smaller size and much smaller corresponding photon counts. Whereas an application to molecules of similar size as tested here should not require much further parameter tuning, such tuning will be required for an application to even smaller or larger proteins, or more realistic forward models including background noise.

Each optimization was run for a predetermined number of steps (as listed in Table S1). At the chosen number of optimization steps app all replicas consistently reached the reference likelihood value. At lower numbers of steps (remapping the parameter schedules) the

convergence was not always successful, with some replicas reaching final structures with reduced likelihood. For a practical application to experimental data with unknown ground truth several replicas should be run to ensure sufficient convergence.

Initialization. The initial bead positions were drawn randomly from spheres of radius 10 Å for Crambin and 15 Å for PDZ-domain and Lysozyme. These radii were chosen such that the resulting balls were smaller then the corresponding ground truth structures, which we have found to work best. Because this initialization is quite far away from the ground truth structures, the initial gradients are very large. Therefore, we started the optimizations with a lower momentum parameter $\beta = 0.8$, which is then scaled up to $\beta = 0.995$ over 1000 steps for Crambin and 200 steps for PDZ-domain and Lysozyme, as can be seen in Figure S1.

Numerical parameters and orientation grids. See Table S1 for numerical parameters used for each test case. The orientations $\mathbf{R}_l$ from eq. (6) where defined in terms of Lebedev grids of order l as in our previous study,[37] by mapping each Lebedev grid point v on S^2 to the geodesic rotation $\mathbf{R}(v)$ that maps v to the standard basis vector $\mathbf{e}_z$, that is, $\mathbf{R}(v)v = \mathbf{e}_z$. Note that v does not correspond to the rotation axis of $\mathbf{R}(v)$, but instead determines the relative direction of the beam axis with respect to the sample.

The Lebedev order and the number of angular orientations n_s (equal to the angular grid dimension) were chosen to correspond to roughly the same rotation angle between nearest neighbor orientations. Due to GPU-hardware constraints the number of angular rotations n_s is restricted to multiples of 32, such that only a rough correspondence is possible. We heuristically estimated the resolution achievable from such a grid to be $r\max(\varphi_1, \varphi_2)$, where r is the maximal radius of the structure to be determined, φ_1 is the spacing between nearest neighbors in the Lebedev grid, and $\varphi_2 = 2\pi/n_s$ is the angular grid spacing. The parameters where then chosen such that this estimated resolution better than at least 2 Å. For the Crambin test case, $r \cdot \max(\varphi_1, \varphi_2) \approx 1.5$ Å, and for PDZ-domain and Lysozyme (which have about the same diameter), $r \cdot \max(\varphi_1, \varphi_2) \approx 2.0$ Å.

The grid points $a_{r,s}$ where uniformly spaced up to $\|a_{r,s}\| = 3\text{Å}^{-1}$. As for the orientations, the required radial grid dimension is largely determined by the sample size, and was determined by trial and error to achieve high enough precision for each test case.

Table S1: Parameters used for each optimization run.

Name	PDB	batch size	Lebedev order	radial grid dim.	angular grid dim.	optim. steps	s_1	s_2	s_3
Crambin	1EJG	200 000	47	60	64	50 000	10	30	20
PDZ-domain	1QAU	50 000	65	60	96	20 000	10	10	20
Lysozyme	148L	50 000	65	120	96	20 000	10	50	20

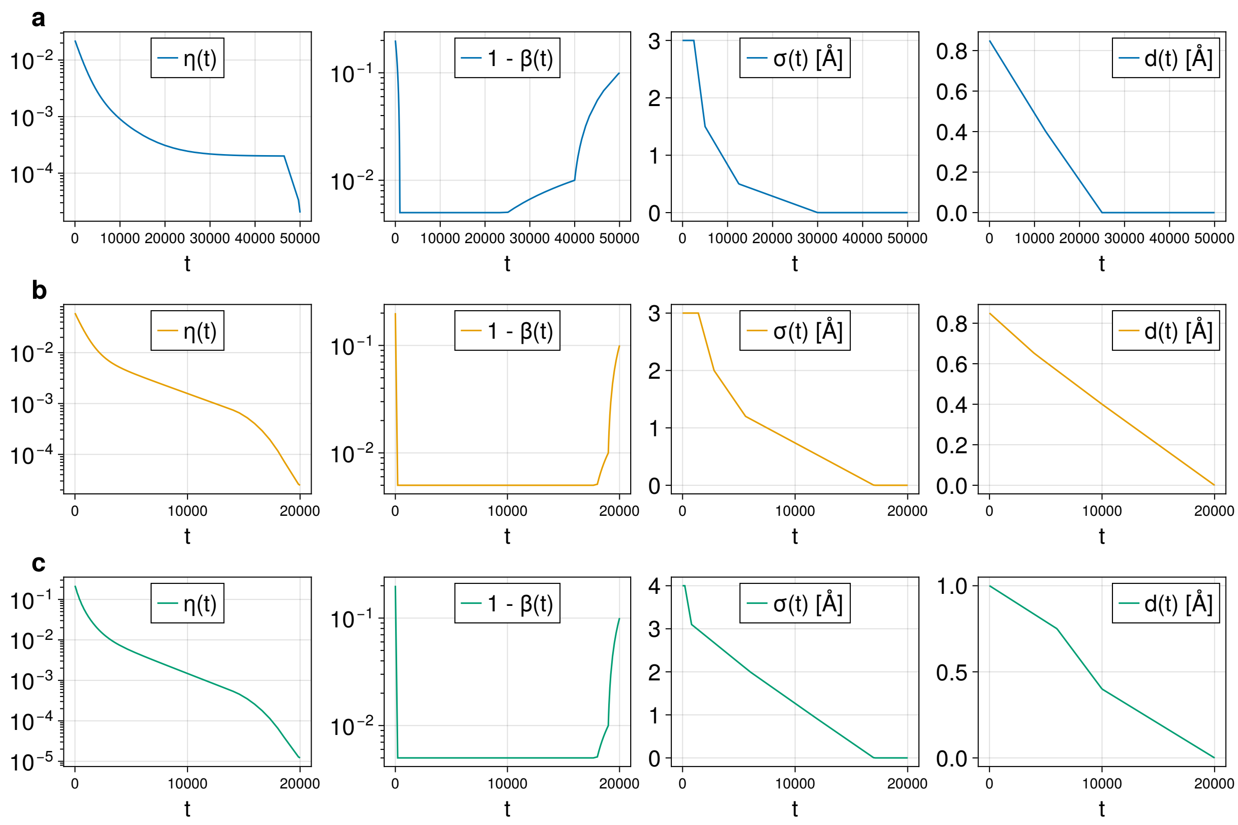

Figure S1: Time dependent parameters $\eta(t)$, $\beta(t)$, $\sigma(t)$, and $d(t)$ used in the optimization runs for **a** crambin, **b** PDZ-domain, and **c** Lysozyme.
